# Lymphatic Migration of Immune Cells

**DOI:** 10.3389/fimmu.2019.01168

**Published:** 2019-05-28

**Authors:** Henry R. Hampton, Tatyana Chtanova

**Affiliations:** ^1^Institute for Systems Biology, Seattle, WA, United States; ^2^Immunology Division, Garvan Institute of Medical Research, Sydney, NSW, Australia; ^3^Faculty of Medicine, St. Vincent's Clinical School, University of New South Wales Sydney, Kensington, NSW, Australia

**Keywords:** lymphatic, migration, neutrophils, T cells, dendritic cells

## Abstract

Lymphatic vessels collect interstitial fluid that has extravasated from blood vessels and return it to the circulatory system. Another important function of the lymphatic network is to facilitate immune cell migration and antigen transport from the periphery to draining lymph nodes. This migration plays a crucial role in immune surveillance, initiation of immune responses and tolerance. Here we discuss the significance and mechanisms of lymphatic migration of innate and adaptive immune cells in homeostasis, inflammation and cancer.

## Introduction

The lymphatic system transports fluids from the periphery back into the circulatory system (1) using a series of open-ended capillaries known as lymphatic vessels (2). This network can be exploited by pathogens to facilitate rapid spread throughout the host (3). To prevent pathogen dissemination and enable a fast targeted immune response, the lymphatic system possesses filter-like structures termed lymph nodes (LNs) (3), where innate immune cells, such as macrophages, neutrophils and dendritic cells (DCs) trap and kill pathogens (3) and activate the adaptive immune response (4).

There are two routes by which immune cells can enter LNs: leukocytes can arrive from the bloodstream by crossing high endothelial venules (HEVs) (5). Alternatively, tissue-resident immune cells can enter afferent lymphatic vessels and migrate to draining LNs (dLNs) (5–8). Cells of the innate immune system including DCs, neutrophils, monocytes as well as adaptive immune leukocytes, such as T and B cells use lymphatic vessels to migrate from tissues into LNs (6–11). Lymphocytes exit LNs *via* efferent lymphatic vessels, and eventually return to the circulatory system by the thoracic duct (12), however, in this review we will focus on the mechanisms and consequences of immune cell migration *via* the afferent lymphatic system.

*In vitro* and *ex vivo* models including adhesion and transmigration assays and analysis of immune cell migration in explanted skin provided important mechanistic insight into leukocyte entry and migration within lymphatic vessels (13–17), while *in vivo* approaches allowed to examine this complex biological process *in situ* (Table 1). Historically, *in vivo* analysis of immune cell migration in afferent lymphatics involved direct transfer of immune cells into the skin, lymphatic cannulation, as well application of fluorescent sensitizers to the skin to label cells and induce inflammation (18). More recently photoconvertible transgenic mice have been utilized to track immune cell migration from the skin and tumors (6, 9, 19–23), while intravital imaging approaches, such as *in vivo* two-photon microscopy enabled direct visualization of immune cell migration in lymphatic vessels (6, 16, 24, 25).

**Table 1:** Methods for investigating immune cell migration *via* afferent lymphatic vessels.

**Approach**	**Advantages**	**Limitations**
Adhesion and transwell assays and explanted skin preparations	•Can be used for chemical and genetic manipulation of cells of interest •Allows to investigate molecular mechanisms of lymphatic migration	•*Ex vivo* or immortalized cells may differ phenotypically and functionally from the same cells *in vivo* •May not replicate all the biological conditions, such as temperatures, pressures, and solute concentrations found *in vivo* •Tissue preparation may alter cellular functions
Direct transfer of purified and labeled donor cells into the skin	•Technically straight forward •Donor cells can be manipulated *ex vivo* •Allows to investigate molecular mechanisms and kinetics of migration	•The isolation and *ex vivo* manipulation of cells may alter cellular phenotypes •Non-physiological cell numbers are used to detect migrating cells •Transferred cells are not native to tissues
Mobilization of tissue immune cells by application of fluorescent tracers/sensitizers	•Can be used to examine migration of endogenous cells in response to inflammation	•Relies on uptake of tracer by cells of interest •Fluorescent label can be taken up by lymph node cells •Induces inflammation
Lymphatic cannulation	•Provides direct insight into the cellular content of normal afferent lymph	•Difficult to perform on small animals •Cannulation may induce inflammation •Anesthesia may alter lymphatic migration
Photolabeling of cells in photoconvertible transgenic mice using UV or violet light to monitor migration of endogenous cells *in vivo*	•Cells can be labeled *in situ* by exposure to light •No *ex vivo* manipulation required •Steady-state and inflammation-induced migration can be accurately quantified	•Difficult to perform in internal organs, requires surgery •Anesthesia may alter lymphatic cell migration •UV light may induce an inflammatory response, however, this response can be reduced if violet light is used to photoconvert
Intravital microscopy to directly visualize immune cells migrating inside lymphatic vessels	•Can be used to directly visualize immune cell migration and interactions with lymphatic vessels in their native environment •Provides information about cellular dynamics	•Requires a dedicated imaging setup •Requires fluorescent reporter mice or adoptive transfer of labeled cells •Anesthesia may alter lymphatic cell migration •Surgery to expose internal organs may cause extensive inflammation

## Lymphatic Migration of Innate Immune Cells

### Dendritic Cells

**Figure 1:**
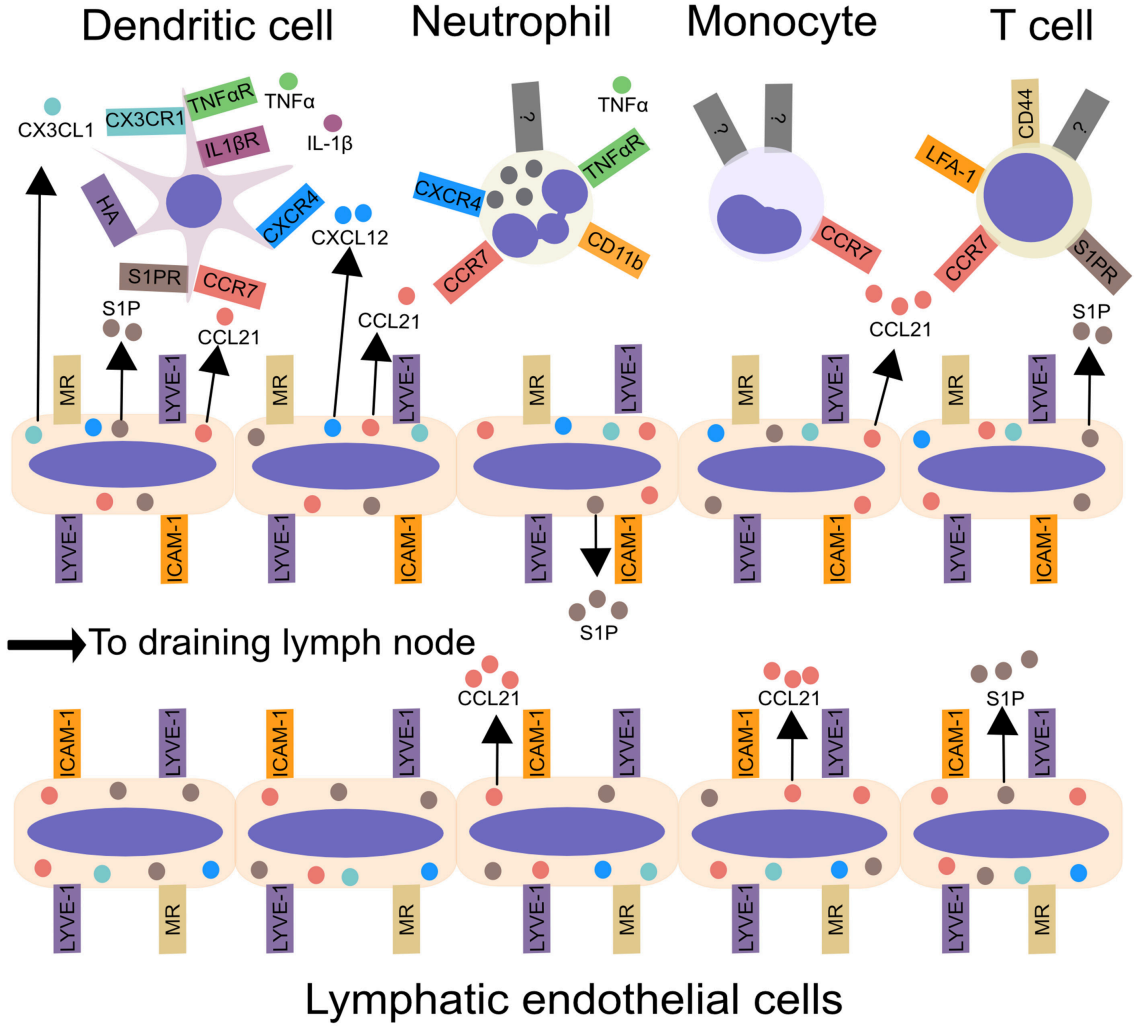
Leukocyte migration from peripheral tissues to draining lymph nodes *via* afferent lymphatic vessels. Inflammatory cytokines, including IL-1β and TNF-α, produced by tissue-resident myeloid cells, enhance DC and neutrophil migration from tissues to lymphatic vessels. Chemokines, such as CCL21, CX3CL1, and CXCL12, synthesized by LECs in the skin control leukocyte migration to lymphatic vessels and aid transmigration into the vessel lumen. In addition to chemokines, lymphatic endothelial cells produce the lipid S1P, which acts upon S1P receptors, to promote the migration of DCs and T cells into lymphatic vessels and aid trafficking to the draining lymph node. Integrins, such as ICAM-1, CD11b, and LFA-1 may promote leukocyte entry into lymphatic vessels and subsequent migration within lymphatics. Interactions between CD44 and MR promote T cell entry into lymphatic vessels. Lastly, LYVE-1 can bind to hyaluronic acid on DCs, and promote DC entry into lymphatic vessels. ICAM-1, Intercellular Adhesion Molecule 1; LFA-1, lymphocyte function-associated antigen 1; LYVE-1, Lymphatic vessel endothelial hyaluronan receptor 1; MR, macrophage mannose receptor; S1P, sphingosine-1-phosphate.

There are two distinct DC populations: plasmacytoid, which produce high amounts of type 1 interferon, and conventional DCs (cDCs) (26). Upon sensing inflammatory stimuli, cDCs enter lymphatic vessels and migrate to LNs (26, 27). They carry antigens (8) and pathogens including viruses (28, 29), spores (30) and bacteria (31–33) from the site of infection to LNs, while DC-mediated transport of innocuous antigens regulates tolerance (34). In dLNs DCs present antigen to CD4^+^ T cells, or cross-present to CD8^+^ T cells, thereby regulating adaptive immune responses (26). DC migration from the periphery has been discussed extensively in several recent reviews (27, 35). Here we provide a brief overview of the mechanisms of DC migration *via* lymphatic vessels (Figure 1).

The most important regulator of DC migration is the chemokine receptor CCR7. Consistent with this, CCR7-deficient DCs show a 90 percent reduction in migration from the periphery in response to inflammatory stimuli (10, 36). An elegant intravital microscopy study demonstrated that CCR7 is required for the LPS-induced directed migration of DCs toward lymphatic vessels and subsequent transmigration (25). DCs also use CCR7 for trafficking to dLNs from the lamina propria (37), lung (34), and skin (23) under homeostatic conditions. Interestingly, CCR7-dependent DC migration decreases lymphatic permeability (38), indicating bi-directional communication between the lymphatic network and immune cells.

Lymphatic vessels express CCR7 ligand, CCL21 (25, 39–41), which is required for DC trafficking from skin to LNs under homeostatic (41) and inflammatory (42) conditions. Imaging studies have provided important insight into the role of CCL21 in DC migration. Firstly, the size of the CCL21 gradient, and distribution of lymphatic vessels, indicates that most skin DCs are able to sense CCL21 gradients (41). Secondly, DCs enter lymphatic vessels at sites of high CCL21 expression (25), suggesting that CCL21 directly regulates entry into lymphatics. Finally, intravital microscopy has revealed that CCL21 also enhances DC migration within lymphatic vessels (40). Collectively, these observations suggest that CCL21 regulates multiple steps in the lymphatic migration of DCs. In contrast, the other CCR7 ligand, CCL19, appears to be dispensable for DC lymphatic trafficking (42).

Inflammatory mediators regulate DC lymphatic migration (26). The cytokines Interleukin-1β (IL-1β) and Tumor Necrosis Factor-α (TNF-α) promote inflammation-induced DC migration to LNs (43–45). Furthermore, the lipid prostaglandin E2 increased CCR7 expression on DCs, augmenting migration towards CCL19 and CCL21 *in vitro* (46). Additional regulators of CCR7-mediated migration include the cell surface molecules CD37, CD38, and CD47 which enhance DC movement toward CCR7 ligands and migration to dLNs (15, 47–50). In contrast, immunosuppressive molecules including IL-10 (51), TGF-β (52, 53) and the anti-inflammatory lipid Resolvin E1 (54) can inhibit DC trafficking.

In addition to CCR7, a number of other chemokine receptor/ligand pairs have been implicated in lymphatic DC migration. CXCR4/CXCL12 and CX3CR1/CX3CL1 enhance DC trafficking from inflamed skin (55, 56). The role of CCR8 is less clear with CCR8-deficient mice displaying reduced lymphatic migration of DCs following an injection of latex beads (57), but enhanced migration of DCs following FITC painting (58), suggesting that CCR8 plays a limited, or stimulus-specific, role in this process.

Intravital imaging and FITC-painting experiments have demonstrated that integrins, and integrin signaling are required for inflammation-induced DC migration to dLNs (14, 59, 60). However, DCs from mice lacking all integrins were able to migrate to LNs when injected into resting skin (61), indicating that integrins are important for DC migration in response to inflammation but dispensable for steady state DC egress. Accordingly, inflammation increases the expression of integrin ligands on lymphatic endothelial cells (LECs) (14). DC-expressed L1 cell adhesion molecule guides transendothelial migration of DCs thereby promoting trafficking to dLNs (17). A recent study demonstrated that interactions between LEC-expressed LYVE-1 and hyaluronan on the DC plasma membrane mediated DC adhesion and transmigration across LECs and subsequent migration to dLNs (13).

Sphingosine-1-phosphate (S1P), a lipid mediator of leukocyte egress from lymphoid organs (62), has been implicated in DC trafficking from the skin and lung (33, 63–65). However, in mice that lack S1P in lymphatic fluid, but not blood, the migration of adoptively transferred DCs to dLNs was comparable to that seen in wild-type mice (66). These results, and the fact that there are five S1P receptors (63), suggest that further experiments are required to uncover the precise role of S1P signaling in DC migration.

In contrast to cDCs, the lymphatic migration of pDCs is poorly understood. While one study reported that adoptively transferred pDCs migrated to dLNs from ovine skin (67), another showed that pDCs were not detected in the lymph of rats (68). However, pDCs transported harmless inhaled antigen from murine lungs to the mediastinal LN where they suppressed T cell activation, suggesting that pDC migration may play a role in preventing inflammation (69).

### Neutrophils

Neutrophils are the first immune cells recruited to sites of inflammation, where they kill pathogens and release mediators that recruit other leukocytes (70, 71). Until recently neutrophils were thought to die at inflammatory foci. However, several groups, including ours, have shown that neutrophils can enter tissue lymphatic vessels and migrate to dLNs from the site of inflammation (6, 72–74).

**Figure 2:**
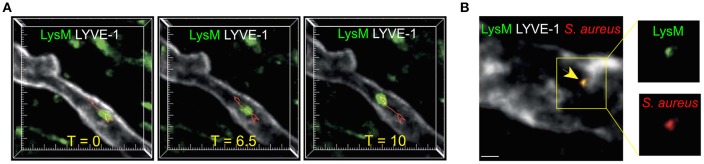
Neutrophil migration in skin lymphatic vessels. **(A)** Two-photon microscopy was used to examine the lymphatic migration of neutrophils in response to *S. aureus*. Images are maximum intensity projections of three-dimensional volumes acquired *via* two-photon microscopy. Lysozyme M^+^ GFP neutrophil (green) migrating inside a lymphatic vessel (LYVE-1, white) is show at three representative time points. Red track indicates neutrophil's path. Tick marks are 10 μm apart. **(B)** Two-photon image of a Lysozyme M reporter mouse skin with a Lysozyme M^+^ (green) neutrophil containing *S. aureus* (red) inside the LYVE-1^+^ lymphatic vessel (white). Scale bar is 10 μm. Figure was adapted from Hampton et al. (6).

Intravital imaging of inflamed mouse skin has enabled direct visualization of neutrophil migration within lymphatic vasculature (Figure 2A) (6, 72). However, in comparison to DCs, the significance and extent of neutrophil lymphatic migration are incompletely understood. Cannulation experiments have demonstrated that inflammation leads to a dramatic increase in neutrophils in ovine afferent lymph (8, 75, 76). Furthermore, neutrophils can transport antigens and microorganisms (Figure 2B) from the site of infection to LNs (6, 8, 77). Accordingly, inhibiting neutrophil lymphatic migration reduced early lymphocyte proliferation (6). Notably, a recent study did not detect substantial lymphatic migration of neutrophils in response to *Staphylococcus aureus* (*S. aureus*) (78). This likely highlights the fact that most neutrophils arrive in dLNs from the circulation *via* HEVs in response to bacteria already in dLNs, while a smaller population of neutrophils migrates directly from the site of inflammation to dLNs *via* afferent lymphatics. However, since neutrophils are the first innate immune cell subset to arrive in the LN from inflamed tissues, and often carry microbes, neutrophil lymphatic migration can exert considerable influence on the subsequent adaptive immune response (6, 77, 79).

Lymphatic migration of neutrophils could potentially be exploited by pathogens to enhance dissemination, since some microorganisms including the bacterium *S. aureus* can survive inside neutrophils (80). Consistent with this, injection of *Leishmania major*-containing neutrophils was sufficient to establish infection in mice, while depleting neutrophils reduced *Leishmania* burden when the pathogen was injected into the skin (81). Neutrophils also transported live *Mycobacterium bovis bacille* Calmette-Guérin from the skin to dLNs (77). In *Toxoplasma gondii* infection neutrophils removed the macrophages that line the subscapular sinus of the LN (82), however, it is not clear whether this favors pathogen control or spread.

CCR7 appears to be less important for neutrophil lymphatic migration than for that of DCs. Although it was required for neutrophil entry into lymphatic vessels in response to TNF-α and Complete Freund's Adjuvant (CFA) (72), and for CFA-driven migration from skin to LNs (83), neutrophil migration from the skin to dLNs in response to *S. aureus* was CCR7-independent (6). This suggests that the requirements for neutrophil trafficking vary depending on the stimulus and additional molecules may play key roles in guiding this migration.

The chemokine receptor CXCR4 regulates neutrophil migration from the bone marrow into the circulation (84) and may also play a role in their lymphatic migration. Inhibiting CXCR4 decreased neutrophil trafficking in response to immune complexes and *S. aureus* (6, 73). However, CXCR4 was not required for neutrophil entry into lymphatic vessels in response to CFA, as revealed by confocal imaging (72), again highlighting differences in neutrophil trafficking in response to distinct stimuli.

The cell surface receptor CD11b, which is involved in neutrophil recruitment from the vasculature into tissues (85), is emerging as a major regulator of neutrophil migration *via* lymphatics since neutrophil migration from inflammatory foci to LNs is substantially reduced when CD11b is inhibited (6, 73, 74). Intravital imaging demonstrated that blocking CD11b or its ligand ICAM-1 impaired neutrophil intraluminal crawling within lymphatic vasculature following CFA injection by reducing neutrophil speed and directionality (72). Likewise, inhibiting CD11b and ICAM-1 reduced neutrophil entry into lymphatic vessels and diminished egress from the skin in response to *Mycobacterium bovis* (74). Lymphocyte Function-associated Antigen (LFA-1), which binds to ICAMs and is involved in neutrophil entry into tissues from the circulation, was required for neutrophil migration *via* afferent lymphatics in response to immune complexes (73) but not *S. aureus* (6).

Inflammatory cytokines may enhance neutrophil entry into lymphatics. TNF-α promoted neutrophil entry and crawling within lymphatic vessels in mouse cremaster muscle (72). However, since inflammatory cytokines also control neutrophil recruitment to sites of inflammation and lifespan, identifying a distinct role for these molecules in neutrophil lymphatic migration requires further investigation.

### Monocytes and Macrophages

Monocytes are circulating leukocytes that phagocytose and kill bacteria and fungi and regulate the activity of other immune cells *via* cytokine release (86–88). They can also differentiate into DC and macrophage subsets (89). Several studies have demonstrated that monocytes egress tissues *via* afferent lymphatic vessels and transport antigen to dLNs (7, 8, 90–92). Once there, monocytes may present and cross-present antigens since a subcutaneous injection of antigen-pulsed monocytes induced the proliferation of antigen-specific CD4^+^ and CD8^+^ T cells (91). The molecular mechanisms controlling monocyte migration *via* lymphatic vessels are yet to be identified. However, CCR7 may be important, since CCR7-deficient LPS-primed monocytes failed to migrate from the footpad to the popliteal LN (91). Accumulating evidence suggests that macrophages can also migrate from inflammatory lesions to dLNs (93–95) and that α1β1 integrin may limit macrophage egress *via* afferent lymphatics (93).

## Lymphatic Migration of Adaptive Immune Cells

### T Cells

T cells possess a rearranged T cell receptor which includes either αβ or γδ polypeptides (96). While αβ T cells are more abundant, γδ T cells are enriched in epithelial and mucosal tissues where they act as the first line of defense against pathogens. One of the main functions of CD8^+^ T cells is to kill infected cells (97), while CD4^+^ helper T cells secrete cytokines and regulate the function of other immune subsets (98). Photoconversion experiments (22), along with lymphatic cannulation (99), have demonstrated that effector, rather than naïve, T cells comprise the majority of lymph migrating T cells under homeostatic (22, 99) and inflammatory conditions (22, 100). This migration plays an important role in immune surveillance and in resolution of inflammation (18, 101–103).

Like DCs, T cells use CCR7 to migrate to LNs under homeostatic and inflammatory conditions. Following antigen challenge, T cells overexpressing CCR7 were preferentially lost from the lung and accumulated in the mediastinal LNs (104), while CCR7-deficient T cells failed to migrate from the footpad to the popliteal LN (11). However, the need for CCR7 in T cell migration might be context dependent, as T cells used CCR7 for trafficking from acute, but not chronically, inflamed skin (100). Tumor-infiltrating T cells could also emigrate to dLNs independently of CCR7 (9).

The lipid S1P, which promotes αβ T cell exit from LNs (62), can also mediate their migration *via* afferent lymphatics. Consequently, antagonizing S1P receptors led to T cell accumulation near skin lymphatic vessels and reduced migration to dLNs (100, 105). LEC-expressed macrophage mannose receptor (MR) and the cell surface molecule CD44, which interacts with MR, promoted T cell lymphatic migration by increasing T cell adhesion to lymphatic vessels (106, 107). Another LEC-expressed protein, CLEVER-1, was also demonstrated to be important for T cell migration *via* afferent lymphatics (108). Additionally, intravital imaging has shown that LFA-1 and its ligand ICAM-1, increased T cell velocity within afferent lymphatic vessels thereby promoting T cell migration to dLNs (16).

T cell egress from tissues to dLNs can either promote inflammatory responses or suppress them. For example, lymphatic migration of Regulatory T (Treg) cells may suppress allograft rejection (103). Likewise, CCR7-deficient Treg cells failed to migrate to the draining LN and accumulated in the skin, reducing skin inflammation during a delayed-type hypersensitivity reaction (101). Conversely, overexpression of CCR7 on antigen-specific Th1-cells enhanced their egress to dLNs and led to faster resolution of the inflammatory response in the skin (102).

Like αβ T cells, γδ T cells can migrate from the skin (23, 95, 109) and tumors (9) *via* lymphatic vessels to dLNs and comprise a large proportion of the cells in bovine lymph (110). Photoconversion experiments have demonstrated that murine γδ T cells can migrate from the skin to dLNs in the absence of CCR7 (23). Similarly, bovine γδ T cells can egress tissues independently of this receptor (111). The consequences of γδ T cell lymphatic migration are poorly understood, though it may enhance CD8^+^ T cell proliferation (23).

## B Cells

Cannulation experiments in sheep (112) and photoconversion experiments in mice (95), suggest that B cells use afferent lymphatic vessels to migrate from tissues to dLNs. The mechanisms of this migration are not yet known, however, at least in chronic inflammation, B cell egress may be independent of S1P and requires CCR7 (100). Similarly to T cells, blocking CLEVER-1 reduced B cell migration to dLNs (108).

## Lymphatic Migration of Immune Cells in Disease

The importance of immune cell migration *via* lymphatics in host defense is illustrated by the observations that mice lacking CCR7 are susceptible to microbial and viral infections (113–115). On the other hand, lymphatic migration of immune cells may also augment autoimmunity since preventing immune cell trafficking from the meninges to the cervical LNs reduced the severity of EAE (116). Furthermore, higher densities of lymphatic vessels in transplanted corneas (117) and kidneys (118) were associated with rejection, while preventing DC migration to the dLN by blocking Vascular Endothelial Growth Factor C (VEGF-C) improved corneal transplantation outcomes (119). Interestingly, in mice, obesity was associated with decreased lymphatic function and reduced immune cell migration to dLNs (120), suggesting that obesity may be linked to decreased immunity. CCR7 as well as its two ligands, CCL19 and CCL21, have been identified in mouse and human atherosclerotic lesions (121), consistent with accumulating evidence of a role for immune cell lymphatic migration in heart disease (122–125).

Lymphatic vasculature also plays a crucial role in tumor immunity by enabling transport of antigens from tumors to dLNs and egress of immune cells (9, 126). Lymphatic vessels can also serve as conduits for tumor cell spread (1). Their dual role in cancer is highlighted by the findings that many tumors overexpress VEGF-C, which promotes the growth and survival of LECs (127, 128) leading to increased LN metastasis (128–130). Furthermore, a recent study demonstrated that IFNγ-induced PD-L1 expression by LECs may dampen anti-tumor immunity by limiting cytotoxic CD8^+^ T cell accumulation in melanoma (131). On the other hand, overexpression of VEGF-C in a mouse melanoma model increased DC migration to dLNs (132). Consistent with an increase in lymphatic migration in cancer, enhanced trafficking of adoptively transferred B and T lymphocytes from footpads to dLNs was observed in melanoma-bearing mice (133).

Similarly to DC migration from the periphery, the DC-dependent transfer of antigen from B16F10 melanoma to the dLN required CCR7. This correlated with an increase in CD8^+^ T cell priming and a reduction in tumor growth (126). Overexpression of TGF-β1 in a model of squamous cell carcinoma reduced DC trafficking to dLNs, which led to an increase in LN metastasis (53). However, since CCR7 and the TGF-β1 receptor are not restricted to DCs (134), other immune cells may also contribute to these effects on tumor growth and metastasis. In contrast to DCs, photoconversion experiments demonstrated that tumor-infiltrating T cells do not require CCR7 to migrate to dLNs *via* afferent lymphatics (9).

## Concluding Remarks

Lymphatic migration of immune cells presents opportunities for control of immune responses in infection and homeostasis. However, with the exception of DCs and T cells, the mechanisms controlling lymphatic migration of immune cells remain poorly understood. New tools, such as photoconversion and intravital imaging are poised to provide novel insight into the migration of previously overlooked immune subsets. A better understanding of the distinct mechanisms guiding lymphatic migration of specific immune subsets may suggest new approaches for treatment of cancer, autoimmunity and excessive inflammation.

## Author Contributions

All authors listed have made a substantial, direct and intellectual contribution to the work, and approved it for publication.

### Conflict of Interest Statement

The authors declare that the research was conducted in the absence of any commercial or financial relationships that could be construed as a potential conflict of interest.
